# Child Melioidosis Deaths Caused by *Burkholderia pseudomallei*–Contaminated Borehole Water, Vietnam, 2019 

**DOI:** 10.3201/eid2808.220113

**Published:** 2022-08

**Authors:** Quyen T.L. Tran, Phuc H. Phan, Linh N.H. Bui, Ha T.V. Bui, Ngoc T.B. Hoang, Dien M. Tran, Trung T. Trinh

**Affiliations:** Vietnam National University, Hanoi, Vietnam (Q.T.L. Tran, L.N.H. Bui, H.T.V. Bui, T.T. Trinh);; Vietnam National Children’s Hospital, Hanoi (P.H. Phan, N.T.B. Hoang, D.M. Tran)

**Keywords:** bacteria, melioidosis, *Burkholderia pseudomallei*, bore water, borehole construction, borehole maintenance, waterborne diseases, Vietnam

## Abstract

Within 8 months, 3 children from 1 family in northern Vietnam died from melioidosis. *Burkholderia pseudomallei* of the same sequence type, 541, was isolated from clinical samples, borehole water, and garden and rice field soil. Boreholes should be properly constructed and maintained to avoid *B. pseudomallei* contamination.

The gram-negative soil-dwelling saprophytic bacterium *Burkholderia pseudomallei* causes melioidosis, a fatal disease highly endemic to Southeast Asia and northern Australia (*1*). Humans can be infected with *B. pseudomallei* via inoculation, inhalation, and ingestion. Rice farmers are at high risk for infection because of their frequent exposure to soil and water, but newborns, children, and older persons also are at risk (*2*,*3*). We report 3 melioidosis deaths among children in northern Vietnam.

## The Study

In November 2019, the Preventive Health Center of Soc Son district in Vietnam reported the deaths of 3 children from 1 family. The first child, a 7-year-old girl, had a high fever and abdominal pain on April 6, 2019. Two days later, she was admitted to a local hospital; after 1 day, she was transferred to St. Paul Hospital in Hanoi, where septic shock was diagnosed. She died on April 9, shortly after admission, before any diagnostic tests were performed. 

On October 27, 2019, the second child, a 5-year-old boy, had a high fever and abdominal pain around the umbilicus. He was admitted to Vietnam National Children’s Hospital in Hanoi on October 28 with diagnosed septic shock. Abdominal and chest radiographs and abdominal ultrasound results were unremarkable. His blood culture grew *B. pseudomallei*, and he died on October 31. 

The third child, a 13-month-old boy, had a high fever and poor appetite on November 10, 2019. According to his grandparents, he had black stool, like his sister and brother. He was admitted to Vietnam National Children’s Hospital; chest radiography results were unremarkable, but *B. pseudomallei* was cultured from his blood sample. He died on November 16. 

We retrieved laboratory findings from all hospitals to which these children were admitted. Results showed leukopenia, neutropenia, thrombocytopenia, and high procalcitonin and C-reactive protein in all children’s blood. Liver dysfunction was diagnosed in all 3 children, but kidney dysfunction was recognized only in the 2 older children. We detected no identifiable risk factors (Table 1).

**Table 1:** Demographic and clinical characteristics and corresponding isolates from 3 children who died of melioidosis caused by *Burkholderia pseudomallei*–contaminated borehole water, Vietnam, 2019*

Characteristics	Case 1			Case 2			Case 3			
Age, y/sex	7/F			5/M			1/M			
Date										
Symptom onset	Apr 6			Oct 27			Nov 10			
Hospital admission	Apr 9			Oct 28			Nov 11			
Death	Apr 9			Oct 31			Nov 16			
Signs and symptoms	High fever, abdominal pain, vomiting, diarrhea with mucous, tachycardia, and cyanosis			High fever, abdominal pain, vomiting, tachypnea, and tachycardia			High fever, poor appetite, mild pitting edema in the feet and hands, tachypnea, and tachycardia			
Underlying disease	Not detectable			Not detectable			Not detectable			
Microbiology										
Blood culture	ND			*B. pseudomallei*–positive			*B. pseudomallei*–positive			
Sequence type	ND			541			541			
Other sample cultures	ND			ND			ND			
Antimicrobial drug treatment	Cefoperazone in the first day; then efoperazone and amikacin on subsequent days			Ceftriaxone, tobramycin, and metronidazole in the first day; then meropenem and levofloxacin on subsequent days			Ceftazidime in the first 2 days; meropenem in the last 3 days			
Imaging at admission										
Chest radiograph	NA			No abnormalities noted			No abnormalities noted			
Abdominal radiograph	NA			No abnormalities noted			NA			
Abdominal ultrasound	NA			No abnormalities noted			NA			
Laboratory findings	Day 1	Day 2		Day 1	Day 2		Day 1	Day 3	Day 4	Day 5
WBC, × 10^9^ cells/L	0.6	0.7		25.2	0.35		10.8	7.5	1.35	1.06
Neutrophils, × 10^9^ cells/L	NA	0.12		22.8	0.05		8.4	4.0	0.76	0.38
Lymphocytes, × 10^9^ cells/L	NA	0.48		1.07	0.29		1.52	3.07	0.50	0.55
Platelets, × 10^9^ cells/L	47	36		272	29		264	172	67	32
Urea, mmol/L	8.9	9.9		NA	9.8		2.2	1.4	3.7	4.1
Creatinine, µmol/L	91	123		NA	124		45	33	55	71
AST, U/L	571	713		NA	602		23	59	185	269
ALT, U/L	226	258		NA	166		10	40	94	73
CRP, mg/L	124	NA		26	148		57	NA	209	158
PCT, ng/mL	NA	>100		NA	>100		9	43	NA	NA

*Data were collected from the St. Paul Hospital and Vietnam National Children’s Hospital, except for the laboratory findings for case 1, which were retrieved from the child’s local hospital. ALT, alanine aminotransferase; AST, aspartate aminotransferase; CRP, C-reactive protein; NA, not available; ND, not done; PCT, procalcitonin; WBC, white blood cell count.

**Figure 1:**
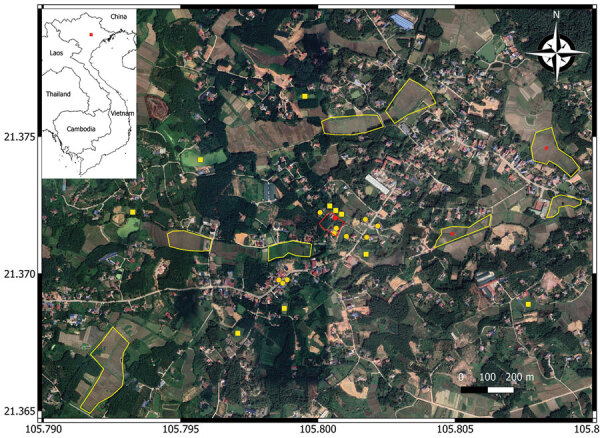
Environmental sampling sites in an investigation of 3 child deaths from melioidosis caused by *Burkholderia pseudomallei–*contaminated borehole water, Vietnam, 2019. The satellite map was created using QGIS software version 3.22.1 (https://www.qgis.org). Red outline indicates the family property where the children lived; red circle is borehole A from which *B. pseudomallei* was isolated. Yellow outlines are rice fields from which soil samples were collected; red stars indicate rice fields that tested positive for *B. pseudomallei*. Yellow circles indicate neighbors’ boreholes and yellow squares indicate neighbors’ ponds from which water samples were collected. Inset map shows Vietnam; red square indicates sampling area.

To trace the source of infection, on November 17, 2019, we visited the family home in the midland region of northern Vietnam (Figure 1). During our active surveillance for melioidosis cases admitted to provincial and tertiary hospitals surrounding Hanoi (*4*), no previous cases had been reported from this area. 

We interviewed the parents and grandparents using epidemiologic questions about all the children’s daily activities inside and outside the house. The family used water supplied from 3 boreholes: 1 for bathing (borehole A), 1 for livestock (borehole B), and 1 for human consumption (borehole C). During our first environmental investigation, we collected samples of front garden soil (n = 7), borehole water (n = 9), and boiled drinking water (n = 1). We performed qualitative culture for *B. pseudomallei*, and all 3 water samples collected from borehole A tested positive (Appendix).

**Figure 2:**
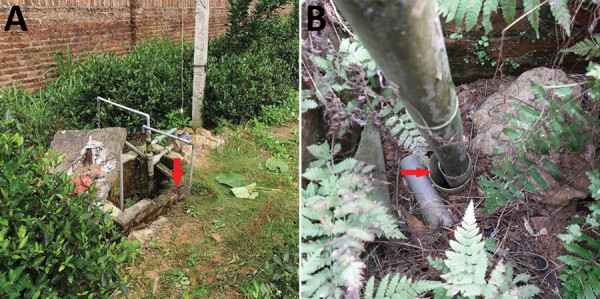
Borehole involved in 3 child melioidosis deaths caused by *Burkholderia pseudomallei–*contaminated borehole water, Vietnam, 2019. A) View of area around borehole. The bore cap is ≈80 cm below the soil surface inside the masonry area. Red arrow indicates cracks in the masonry construction that might enable rainwater and soil particles to drain into the borehole area. B) View from above the borehole. Red arrow indicates the unsealed, opened gap around the borehole, which likely enabled rainwater and soil particles to drain into the groundwater during the rainy season.

We revisited the home on November 23, 2019, and asked the family about the history of borehole A. In brief, the borehole was drilled in 2010. In 2015, the family reconstructed the back garden and added a new soil layer, resulting in the bore cap being ≈80 cm below the soil surface (Figure 2, panel A). At the end of 2018, the foot valve in the suction pipe of the dynamic electric pump was damaged, and the bore cap was not sealed after the damage was repaired (Figure 2, panel B). We suspected rainwater and surface soil particles contaminated with *B. pseudomallei* drained into the groundwater via the opened borehole. To test this hypothesis, we conducted a second round of environmental sampling, focusing on borehole A and the nearby surface soil. We collected 26 borehole water and 46 garden soil samples. Within a 1-km radius of the home, we also collected 39 water samples from other boreholes, 30 surface water samples from 10 ponds, and 40 soil samples from 8 rice fields (Figure 1; Appendix). 

We found 26 (100%) water samples collected from borehole A and 27 (58.7%) garden soil samples from 8 (80%) sampling points near the borehole were *B. pseudomallei*–positive by qualitative culture. These findings supported our hypothesis that *B. pseudomallei* from surface soil might have contaminated the groundwater through the unsealed bore cap during the rainy season, which starts in April and coincided with the first child’s illness and death. Another 5 (12.5%) soil samples from 2 (25%) rice fields also tested *B. pseudomallei*–positive. Quantitative culture showed that the median *B. pseudomallei* count was 406 CFU/g (range 12–746 CFU/g) in soil (Appendix). Of 26 water samples collected from borehole A, 2 (7.7%) grew *B. pseudomallei* on the initial agar plates and had a median *B. pseudomallei* count of 2 CFU/mL (Table 2).

**Table 2:** Culture results and genotype data from environmental samples in a study of 3 child melioidosis deaths caused by *Burkholderia pseudomallei–*contaminated borehole water, Vietnam, 2019*

Sample type, date	No. samples	No. sampling points†	Qualitative culture		Median quantitative count, CFU (range)	No. isolates selected for MLST‡	ST
			No. (%) positive samples	No. (%) positive sampling points†			
Sampling 1, 2019 Nov 17							
Front garden soil	7	7	0	0	NP	NA	
Water from borehole A	3	1	3 (100)	1 (100)	2§	3	541
Water from borehole B	3	1	0	0	NP	NA	NA
Water from borehole C	3	1	0	0	NP	NA	NA
Boiled drinking water	1	1	0	0	NP	NA	NA
Sampling 2, 2019 Nov 23							
Back garden soil near borehole A	46	10	27 (58.7)	8 (80)	406 (12–746)§	6	541
Rice field soil	40	8	5 (12.5)	2 (25)	ND	5	541
Water from borehole A	26	1	26 (100)	1 (100)	ND	4	541
Water from borehole B	3	1	0	0	NP	NA	NA
Water from borehole C	3	1	0	0	NP	NA	NA
Water from neighbors’ borehole	33	11	0	0	NP	NA	NA
Water from ponds	30	10	0	0	NP	NA	NA

*CFU, colony forming unit; MLST, multilocus sequence typing; NA, not applicable; ND, not detected; NP, not performed; ST, sequence type. †Sampling points refer to garden, borehole, field, and pond sites. ‡We selected 20 *B. pseudomallei* isolates for sampling; 2 patient isolates are not shown here. §*B. pseudomallei* colonies were countable only in 2 borehole water samples and 5 garden soil samples (Appendix). In water samples CFU/mL; in soil samples CFU/g.

We selected 20 *B. pseudomallei* isolates for multilocus sequence typing (MLST) (*5*): 7 from borehole A, 6 from back garden soil, 5 from rice field soil, and 2 from blood samples from cases 2 and 3. MLST showed an identical sequence type (ST), 541, among all samples (Table 2).

## Conclusions

*B. pseudomallei* is ubiquitously distributed in soil and surface water throughout the tropics, including in Asia, the Pacific Islands, sub-Saharan Africa, and Latin America, where boreholes are the most common water supply in the rural areas (*1*,*6*,*7*). In addition to other waterborne infections (*7*), untreated water supplies have been implicated in previous human *B. pseudomallei* infections (*8*–*10*). *B. pseudomallei* also was isolated from the compacted earth floor under the bathing tub of a woman who died from septicemic melioidosis in Brazil (*11*). 

Studies in Australia and Thailand detected diverse STs among *B. pseudomallei* isolates from an unchlorinated bore water site and a single soil sample (*12*,*13*), but our analysis revealed a single ST in the borehole, nearby garden, and surrounding rice fields. Because all 3 infections occurred in children, we believe *B. pseudomallei* transmission likely occurred through ingestion of contaminated water during bathing, especially considering that the 13-month-old boy was not in contact with garden or rice field soil. Ingestion also could explain the gastrointestinal symptoms the children exhibited.

*B. pseudomallei* ST541 has been reported from human melioidosis cases in northern Vietnam (*3*) and has only been described from southeast Asia thus far. During previous surveillance (*4*), we found other ST541 isolates in clinical and environmental samples from north and north-central Vietnam. An ST541 isolate available in a public MLST database (https://pubmlst.org/organisms/burkholderia-pseudomallei; accessed 2021 Dec 8) was from a human case in Hainan, China, which is close to the area of Vietnam where these 3 melioidosis deaths occurred. From our clinical data retrieval (*3*,*4*), 5 of 8 patients infected with *B. pseudomallei* ST541 died, which could mean ST541 is more virulent than other STs, but further data are needed. 

From the epidemiologic investigation and field study at the family home, we became aware of the construction and maintenance of the borehole, which had an unsealed cap and an open borehole below the soil surface. The unsealed borehole probably enabled *B. pseudomallei* from surface soil to contaminate groundwater during rainfall. Other studies have reported higher rates of gastrointestinal pathogens in water from boreholes with unsealed annuli (*14*,*15*). Therefore, persons using boreholes in countries where melioidosis is endemic should ensure proper construction and maintenance to avoid contamination with *B. pseudomallei* and other pathogens from surface soil. 

Appendix Additional information on 3 child deaths from melioidosis caused by *Burkholderia pseudomallei*–contaminated borehole water, Vietnam, 2019
