# Supplementary material for: Child Melioidosis Deaths Caused by Burkholderia pseudomallei–Contaminated Borehole Water, Vietnam, 2019
Source: Emerg Infect Dis. 2022 Aug;28(8):1689–93. doi: 10.3201/eid2808.220113 (PMC9328891; doi:10.3201/eid2808.220113)
Supplement: Appendix — Additional information on 3 child deaths from melioidosis caused by Burkholderia pseudomallei–contaminated borehole water, Vietnam, 2019 [file 22-0113-Techapp-s1.pdf]

Article DOI: https://doi.org/10.3201/eid2808.220113

Child Melioidosis Deaths Caused by *Burkholderia pseudomallei*–Contaminated Borehole Water, Vietnam, 2019

Appendix

Materials and Methods

Soil Sampling

A primary environmental investigation was conducted at the family property on November 17, 2019. Seven soil samples at a depth of 10 cm were collected at different sampling points of the front garden. Nine borehole water (3 samples from each borehole) and one boiled drinking water samples were also collected.

A secondary environmental sampling was conducted on November 23, 2019. At borehole A, the electric pump was turned on, and 26 bore water samples were collected every five minutes (from 0 to 120 minutes) and at 240 minutes. Forty-six soil samples from 10 sampling points near borehole A in the back garden were also collected, and the distance between each sampling point was ≈10 m. At each point, the soil samples were collected at depths of 10, 20, 30, 40, and 50 cm, except for 2 points where only soil samples at depths of 10, 20, and 30 cm were collected. Additionally, 39 bore water samples were collected from the other two boreholes on the family property and 11 boreholes in the neighborhood (3 samples from each borehole). Thirty surface water samples from 10 surrounding ponds (3 samples from each pond) were collected. Forty soil samples from eight rice fields were collected at a depth of 30 cm (5 samples from each rice field) (Figure 1).

Qualitative Culture of *B. pseudomallei*

Detection of *B. pseudomallei* from water and soil samples was performed using a two-step enrichment approach (*1*). In brief, a 10-gram soil sample was added to 50 mL tubes containing 20 mL of TBSS-C50 broth. After vigorous vortexing, the tubes were statically

incubated at 40°C for 2 days. Subsequently, 1 mL of the culture supernatants were transferred to new tubes containing 9 mL of MB broth. After static incubation at 40°C for 4 days, the enriched culture supernatants were streaked out on Ashdown agar plates. The plates were then incubated at 40°C for 4 days and examined every day. Based on morphological characteristics, suspected colonies of *B. pseudomallei* were picked up, and the bacterial identification was confirmed using the *B. pseudomallei*-specific real-time PCR assay targeting the TTSS1 gene (*2*). The bacterial isolates were stored at −70°C in Luria-Bertani broth containing 20% glycerol for further genotype experiment.

For water samples, 100 mL of water sample was centrifuged at 5,000 rpm for 30 min, and the supernatants were decanted to obtain the water sediments. Then, 20 mL TBSS-C50 broth was added to the tubes, and the culture approach for *B. pseudomallei* was performed, as described above.

Quantitative Culture of *B. pseudomallei*

The bacterial count was only performed on water or soil samples positive for *B. pseudomallei* by the quantitative culture. In brief, a 10-gram soil sample was added to a 250-mL Erlenmeyer flasks containing 20 mL of distilled water. The soil was dispersed by shaking at 160 rpm for 2 h at room temperature. The flasks were left for 30 min to allow the soil particles settle. Then 100 µL of the upper layer suspension and its serial 10-fold dilutions were plated out on the Ashdown agar plates. After incubation at 40°C for 4 days, the suspected *B. pseudomallei* colonies were counted, and the CFU/g of soil was calculated, as previously described (*3*).

For the water samples, 500 µL of borehole A water was plated out on the Ashdown agar plates. After incubation at 40°C for 4 days, the suspected *B. pseudomallei* colonies were counted, and the CFU/mL of water was calculated.

Physiochemical Parameters of Soil and Water Samples

Compared with *B. pseudomallei*–negative borehole water samples, physicochemical parameters showed water samples from borehole A had low pH and high nitrate, iron, total suspended solids, and total organic carbon (Appendix Table 1). *B. pseudomallei*–positive soil collected in the garden near borehole A had much lower electrical conductivity but much higher total potassium oxide and aluminum levels than *B. pseudomallei*–negative soil samples (Appendix Table 2).

References

1. Trinh TT, Assig K, Tran QTL, Goehler A, Bui LNH, Wiede C, et al. Erythritol as a single carbon source improves cultural isolation of *Burkholderia pseudomallei* from rice paddy soils. PLoS Negl Trop Dis. 2019;13:e0007821. PubMed https://doi.org/10.1371/journal.pntd.0007821

2. Novak RT, Glass MB, Gee JE, Gal D, Mayo MJ, Currie BJ, et al. Development and evaluation of a real-time PCR assay targeting the type III secretion system of *Burkholderia pseudomallei*. J Clin Microbiol. 2006;44:85–90. PubMed https://doi.org/10.1128/JCM.44.1.85-90.2006

3. Trung TT, Hetzer A, Topfstedt E, Göhler A, Limmathurotsakul D, Wuthiekanun V, et al. Improved culture-based detection and quantification of *Burkholderia pseudomallei* from soil. Trans R Soc Trop Med Hyg. 2011;105:346–51. PubMed https://doi.org/10.1016/j.trstmh.2011.03.004

Appendix Table 1. Physicochemical parameters of water samples from 1 *Burkholderia pseudomallei*–contaminated borehole and 13 other boreholes investigated in the deaths of 3 children from melioidosis, Vietnam, 2019*

Physicochemical parameters, mg/mL	Contaminated borehole	Other boreholes
pH	3.91	6.13 (3.98–7.19)
Total suspended solids	2.47	1.35 (0.85–2.67)
Ammonium	ND	0.14 (ND–0.4)
Nitrate	4.80	3.08 (0.22–4.54)
Phosphate	ND	0.21 (ND–0.23)
Iron	0.34	0.19 (0.11–0.30)
Total organic carbon	4.70	3.31 (2.50–4.20)
Chemical oxygen demand	6.00	5.50 (4.00–8.00)
Biologic oxygen demand	1.00	0.75 (0.00–2.00)

*Data represent mean (range); ND, not detected.

Appendix Table 2. Physicochemical parameters of soils collected from the garden and rice fields investigated for *Burkholderia pseudomallei* contamination in the deaths of 3 children from melioidosis, Vietnam, 2019*

	Garden soil			Rice field soil		
Physicochemical parameters	Positive, n = 14	Negative, n = 4	p value	Positive, n = 4	Negative, n = 10	p value
pH_{KCl}	3.73 ± 0.16	3.59 ± 0.11	0.139	4.08 ± 0.24	4.46 ± 0.37	0.087
Moisture content, %	14.30 ± 2.07	16.1 ± 2.52	0.167	18.10 ± 0.15	16.50 ± 3.6	0.395
Electrical conductivity, µS/cm	75.48 ± 25.93	112.60 ± 42.92	0.043	105.50 ± 53.17	98.25 ± 44.73	0.798
Organic carbon, %	0.99 ± 0.19	0.98 ± 0.47	0.935	0.76 ± 0.40	0.66 ± 0.39	0.673
Total nitrogen, %	0.06 ± 0.01	0.05 ± 0.03	0.458	0.05 ± 0.02	0.05 ± 0.02	0.741
Total P_2O_5, %	0.05 ± 0.01	0.06 ± 0.04	0.385	0.07 ± 0.03	0.06 ± 0.03	0.633
Iron, g/Kg	2.18 ± 0.52	2.46 ± 0.60	0.368	2.15 ± 1.31	1.96 ± 2.46	0.889
Total K_2O, %	1.48 ± 0.49	0.57 ± 0.28	0.003	0.96 ± 0.17	0.72 ± 0.26	0.115
Aluminum, g/Kg	4.75 ± 0.59	3.95 ± 0.49	0.026	2.27 ± 1.35	1.94 ± 0.80	0.571

*Data represent mean ± SD; p values were obtained from *t*-test. K_2O, potassium oxide; KCl, potassium chloride; P_2O_5, phosphorus pentoxide; S, siemens.
